# A Narrative Review of Diabetes Intervention Studies to Explore Diabetes Care Opportunities for Pharmacists

**DOI:** 10.1155/2016/5897452

**Published:** 2016-05-10

**Authors:** Shamala Ayadurai, H. Laetitia Hattingh, Lisa B. G. Tee, Siti Norlina Md Said

**Affiliations:** ^1^Curtin University, School of Pharmacy, Kent Road, Bentley, Perth, WA 6102, Australia; ^2^Ministry of Health, Hospital Sultanah Aminah, Jalan Persiaran Abu Bakar Sultan, 80100 Johor Bahru, Johor, Malaysia

## Abstract

*Background*. We conducted a review of current diabetes intervention studies in type 2 diabetes and identified opportunities for pharmacists to deliver quality diabetes care.* Methods*. A search on randomised controlled trials (RCT) on diabetes management by healthcare professionals including pharmacists published between 2010 and 2015 was conducted.* Results and Discussion*. Diabetes management includes multifactorial intervention which includes seven factors as outlined in diabetes guidelines, namely, glycaemic, cholesterol and blood pressure control, medication, lifestyle, education, and cardiovascular risk factors. Most studies do not provide evidence that the intervention methods used included all seven factors with exception of three RCT which indicated HbA1c (glycated hemoglobin) reduction range of 0.5% to 1.8%. The varied HbA1C reduction suggests a lack of standardised and consistent approach to diabetes care. Furthermore, the duration of most studies was from one month to two years; therefore long term outcomes could not be established.* Conclusion*. Although pharmacists' contribution towards improving clinical outcomes of diabetes patients was well documented, the methods used to deliver structured, consistent evidence-based care were not clearly stipulated. Therefore, approaches to achieving long term continuity of care are uncertain. An intervention strategy that encompass all seven evidence-based factors will be useful.

## 1. Introduction

Diabetes contributed to 1.2 million worldwide deaths in 2012 [1]. In people with diabetes, approximately 50–80% of mortality is attributed to cardiovascular disease [2]. Diabetes is the leading cause of kidney failure [3] and also contributes to one percent of global blindness [4]. In terms of healthcare burden it was estimated that in 2010 diabetes contributed to 4–7 billion United States Dollar (USD) in health expenditure in Australia, USD 7–15 billion in the United Kingdom (UK), and USD 197–344 billion in the United States of America (USA) [5].

As the incidence of diabetes and health burden continues to rise [3] a new approach in diabetes management is imminent. The overall aim of diabetes care is to improve patients' quality of life, prevent early death and reduce the burden of disease [6]. Nevertheless, diabetes is a complex disease as there is a need to address multiple factors in order to achieve quality diabetes care. Addressing multiple factors is referred to as* multifactorial intervention* or* multifactorial treatment *and has been described in previous studies [7–13].

This is a narrative review of multifactorial intervention studies from selective literature and explores potential opportunities for pharmacists to deliver quality diabetes care in patients with type 2 diabetes. Findings from this review are useful in addressing current practice challenges.

*Aim of Narrative Review*. The aim of this research is to review current diabetes management practices targeted towards improved diabetes control and prevention of diabetes related complications. The objectives are to

1. critique diabetes studies in terms of diabetes guidelines,
2. determine intervention methods used by healthcare professionals involved in diabetes care with a particular focus towards diabetes intervention services by pharmacists,
3. identify key areas where multifactorial interventions are lacking and explore opportunities for pharmacists in diabetes care.

## 2. Method

Keywords used in database searches were “diabetes”, “pharmacist”, “intervention” or “tool”, and “randomised controlled trial” (RCT). Databases used included the Cochrane Library, PubMed, Medline (Web of Science), ProQuest, Scopus, and Medline Ovid. Searches were limited to articles in the English Language, published between January 2010 and August 2015 and included both type 1 and type 2 diabetes. The findings from the search are presented as a narrative review.

## 3. Results and Discussion

### 3.1. Multifactorial Diabetes Care

There were several landmark trials that provided significant evidence that led to improved diabetes management outcomes, namely:

1. The United Kingdom Prospective Diabetes Study (UKPDS) carried out in the UK [14].
2. The Action in Diabetes and Vascular Disease: Preterax and Diamicron MR Controlled Evaluation (ADVANCE) study carried out in 20 countries throughout Asia, Australia, Europe, and North America [15].
3. The Veterans Affairs Diabetes Trial (VADT) in the USA [16].
4. The Diabetes Control and Complications Trial (DCCT) in the USA [17].
5. The Action to Control Cardiovascular Risk in Diabetes (ACCORD) study carried out in the USA and Canada [18].

Table 1 is a summary of these trials. Findings from the trials provided evidence that three main factors need to be addressed to achieve therapeutic targets and consequently prevent diabetes complications, namely, glycaemic, blood pressure (BP), and cholesterol control.

**Figure 1:**
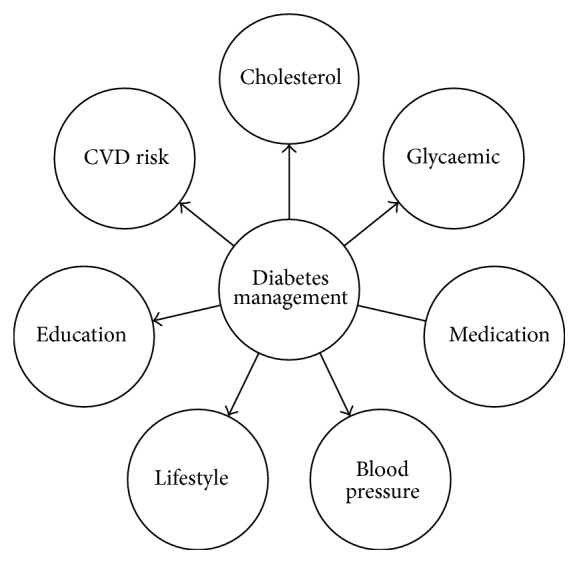
Summary of the seven evidence-based factors required in diabetes management.

These factors have been incorporated into clinical practice guidelines (CPGs) on diabetes management from Australia, Europe, the UK, and the USA. In addition, CPGs from these countries recommend management of four other factors in diabetes management. These are medication management, lifestyle, education, and cardiovascular risk management. These seven diabetes factors are summarised in Figure 1 and discussed in the following paragraphs.

*(1) Glycaemic Control*. Diabetes guidelines recommend a target HbA1c (glycated haemoglobin) of 7% or less. Glycaemic control should aim to reduce HbA1c by 1% or more for patients whose HbA1c is more than 7%, as this can lead to significant reduction in microvascular complications, as was shown in the UKPDS trial [14]. The results from the landmark trials highlight several factors that need to be emphasised to prevent hypoglycaemia such as individualised glycaemic targets, educating patients on hypoglycemia awareness, self-monitoring of blood glucose levels, adjusting therapy, and changing to treatment that causes low risk of hypoglycemia [20–23].

*(2) BP Control*. Blood pressure less than 150/85 mmHg has demonstrated a reduction of microvascular and macrovascular complications [14]. The American Association of Clinical Endocrinologists and American College of Endocrinology CPGs on diabetes strongly suggests a target BP of less than 140/80–90 mmHg and recommended an update from the previous target of less than 130/80 mmHg [24]. However Australian guidelines recommend a target of 130/80 mmHg or lower and 125/75 mmHg for diabetes patients with proteinuria. Diabetes guidelines from Australia and the USA advise on the need to reduce sodium intake, increase potassium intake, and moderate alcohol consumption [25, 26]. These guidelines recommend prescribing, unless contraindicated, an Angiotensin Converting Enzyme Inhibitor (ACEI) or Angiotensin Receptor Blocker (ARBs) as the preferred antihypertensive.

*(3) Cholesterol Control*. Guidelines from Australia, the UK, and USA stress the importance of use of a lipid-regulation medication such as a statin unless contraindicated, to reduce the risk of developing cardiovascular disease (CVD). Although there is increased risk of developing diabetes with statin use [27, 28], several meta-analysis on randomised trials show evidence of increased benefit of statins in terms of reduction in cardiovascular risk [29, 30]. The current safety advice from the USA Food and Drug Administration [31] and Australian Diabetes Guidelines [25] outlines the benefits of statins in preventing cardiovascular events over the increased glycaemia risks.

*(4) Medication Management*. Medication management requires that each patient's medicine related needs be addressed to achieve target therapy outcomes. Pharmacists play a main role in medication management that involves identifying, resolving, and preventing medication-related problems [32]. Medication-related problems include inappropriate medication, incorrect or inappropriate dose, medicine interactions, adverse medicine reactions, and unnecessary medicine use [32].

Addressing patients' medication-related problems facilitates achievement of treatment goals, as documented in a study of 2620 patients in the USA seen by pharmacists [33]. In a similar randomised prospective study of 107 Latino patients in the USA, followed up for two years, adherence to medication was the strongest predictor of reaching the target HbA1c [34]. Reducing hypoglycemia episodes has also been associated with increased patient adherence and satisfaction with medication [35]. Several studies suggest that diabetes patients who are adherent in taking their medication reduce overall healthcare burden, even though this could mean an increase in medication costs [36–40].

*(5) Lifestyle*. Lifestyle factors such as diet intervention, exercise, smoking cessation, moderation of alcohol consumption, and stress reduction contribute to achievement of glycaemic control [41, 42]. Intensive lifestyle interventions resulted in reduction of more than 5% weight loss and the loss was maintained at the fourth year in the Look AHEAD (Action for Health in Diabetes) study [43]. However, there is lack of intervention studies on other lifestyle issues such as foot care despite neuropathy being a major diabetes related complication. In 2005, there were about 10,000 hospital admissions for diabetes related foot ulcers in Australia resulting in lower limb amputations [44]. In the USA, the annual cost of diabetes foot ulcers is USD9-13 billion in addition to other diabetes costs [45].

*(6) Education*. A Malaysian study showed that one of the barriers to achieving good glycaemic control includes lack of understanding and knowledge of diabetes [46]. Educating diabetes patients on the management of their disease can significantly improve glycaemic [47–50], BP [49] and cholesterol control [48, 51], physical activity [49–51], dietary management [51], and medication understanding and adherence [49, 50].

*(7) CVD Risk Prevention Strategies*. Guidelines from Europe, the UK, and USA suggest aspirin therapy (75 mg–162 mg/day) as primary preventative strategy for increased CVD risk (10 year risk >10%) [24, 52, 53]. Cardiovascular disease risk can be estimated using risk prediction formulae such as the Framingham Risk Score and the UKPDS tool for diabetics. In Australia, the absolute CVD disease risk chart/calculator was developed using the Framingham risk equation [54]. USA guidelines recommend the use of the Framingham risk score. The Framingham risk score calculates percentage of CVD risk in 10 years using a patient's information on age, family history of CVD, gender, total cholesterol level, HDL cholesterol level, whether he/she is a smoker, has diabetes, or has systolic BP level, and whether the patient is treated for high blood pressure [55].

### 3.2. Practice Guidelines and Multifactorial Intervention Studies

Diabetes practice guidelines aim to achieve a range of outcomes such as the reduction of microvascular and macrovascular complications, improvement in quality of life (QOL), and prevention of premature mortality. Reductions in several diabetes complications such as kidney failure and amputation were observed as more patients received guideline-adherent therapy [56]. However, there is evidence that guidelines are not always being followed in clinical practice. In a recent cross-sectional study, it was found that among 650 Malaysian outpatients, 32.1% of diabetes patients with hypertension were not on an antihypertensive such as an ACEI or ARB as per guideline recommendations, although these patients had no contraindications to these antihypertensives [57]. A similar study conducted among 430 Australian diabetes patients found evidence-based practice gaps especially in the prescribing of antihypertensive and lipid lowering medications [58].

The multifactorial interventions described in diabetes studies often do not encompass all seven factors addressed in the diabetes guidelines. Diabetes intervention studies led by healthcare professionals other than pharmacists indicated improvements in patients' outcomes. However, there are inconsistencies in terms of the number of diabetes factors being addressed. While some studies emphasised self-management [59–62], others focused only on diet [41] or adherence [63]. Some studies evaluated motivational interviewing to promote behavioural changes and belief among diabetes patients which resulted in improved glycaemic control, adherence, and lifestyle changes [64–69]. Interventions which focused on four factors, namely, nutrition, blood glucose monitoring, medication taking, and lifestyle improved HbA1c and health related QOL [12, 70, 71]. These studies are summarised in Table 2. However, data on the number of patients seeking emergency treatment or who had adverse events in comparison to the usual care group were not always mentioned. Adverse events such as hypoglycemia are not uncommon among diabetes patients, as reported in a meta-analysis and prospective study carried out in seven countries [35, 72].

### 3.3. Pharmacist Led Medication Management Services

The CPGs on diabetes from Australia, Europe, the UK, and USA suggest multiprofessional teamwork when managing patients [24, 25, 52, 53, 73]. One such approach is pharmacist led medication management services (MMS). MMS involves pharmacists working in collaboration with other healthcare professionals such as doctors or endocrinologists, diabetes nurses, and dietitians to improve patient outcomes and health related QOL which could reduce visits to general practitioners (GPs) and hospitalisation rates [74]. MMS pharmacists assess patients' medication to identify, resolve, and prevent medication-related problems. A care plan with recommendations to the physician or practitioner is developed to optimize medication and achieve goals of therapy. Patients are then followed up by the MMS pharmacist to monitor outcomes. MMS studies carried out in different parts of the world suggest that pharmacists in collaboration with other healthcare professionals can make significant improvements in therapy outcomes. Previous studies have reported improvement in HbA1c levels [47, 75–80], reduction in CVD risk score [77, 81], reduction in cholesterol levels [82, 83], and improvement in lifestyle factors [75, 83, 84]. These studies are grouped and summarised according to similar intervention strategies in Table 3.

These studies managed to reduce HbA1c between 0.6% and 1.8%. In accordance with the ACCORD trial it was demonstrated that each 1% reduction in HbA1c reduced 21% of diabetes related endpoints such as macrovascular complications, 21% of deaths associated with diabetes, 14% risk of myocardial infarction, and 37% of microvascular complications [94].

In addition, several studies incorporating QOL on seven attributes, namely, vision, hearing, speech, ambulation, dexterity, emotion, and cognition, reported positive results [76, 80, 92]. QOL measurement is widely accepted as an important goal in health interventions [32] and these studies provide evidence for pharmacists' value in improving both clinical and QOL outcomes for diabetes patients.

### 3.4. Limitations in Medication Management Services

Findings from the Cochrane Collaboration suggested that pharmacist-provided patient oriented services may improve glycaemic control, BP, cholesterol, and QOL of diabetes patients, suggesting that pharmacists' services may reduce visits to GPs and hospitals [74]. Despite this, a 2014 National Centre for Health Statistics report from the USA Department of Health and Human Services found an increase in GP visits made by diabetes patients [95]. The report, however, did not stipulate the reasons for these increases. Several issues could have contributed such as failure to address all seven factors in diabetes management, lack of patient contact with pharmacists, pharmacists' restricted access to patient medical notes, pharmacists' lack of experience in medication management, increasing workload for pharmacists, and sustainability of methods used in medication management services.

Pharmacist RCTs on medication management services do not provide evidence of the seven diabetes management factors that should be addressed in diabetes care as these factors were not consistently incorporated in patient interactions. Despite some studies showing more than 1% reduction of HbA1c, other studies which used the same intervention method showed less reduction or in some cases no difference. The range of HbA1c reduction in RCTs which focused on three or less of the seven factors were 0.8% to 1.0% [78, 83, 85, 90] while RCTs which incorporated four to five factors produced a reduction of 0.6% to 1.7% [47, 75, 80, 82, 84, 86]. In studies which incorporated six to seven evidence-based factors, the HbA1c range improved by 0.5% to 1.8% [77, 79, 91].

It is uncertain if other variables are responsible for these outcomes, for instance, frequent face-to-face contact with patients. RCTs mentioned in Table 3 included frequent visits to the pharmacy every two to four weeks. These studies reported a HbA1c reduction which ranged between 0.5% and 1.7%. However these intervention groups may have had positive outcomes due to the regular contact with the pharmacist and not due to the nature of the intervention itself, as suggested by several studies [96, 97]. Therefore, patients who show less commitment to intervention programs may obtain less benefit. In addition, it is uncertain whether positive clinical outcomes continue after patients' face-to-face management ends.

The methods used to assess the patients' adherence towards medications were not clearly defined in some studies [78, 80, 82, 91] while other studies used varied assessment methods [79, 85–87]. These unstandardised methods could have contributed to the difference in patient outcomes. The assessment methods mentioned in these RCTs were the ASK-20 (Adherence starts with knowledge), prescription refill rates and self report, eight-item modified Morisky adherence assessment score, and the four-item Morisky adherence assessment score.

Lack of access to patients' medical notes and laboratory data may be a barrier for community pharmacists to provide quality medication management compared to pharmacists in hospital settings. In a study conducted in a GP clinic in Australia where pharmacists had access to patient's medical data the results showed increased medication adherence and improved patient satisfaction [98]. The importance of electronic health records in improving healthcare delivery has prompted the USA government to pass legislation to better integrate information technology into healthcare delivery in 2009 [99]. This enabled community pharmacists providing medication management services in the USA to access patients' medical records including information about medications, laboratory, and radiology results. Nevertheless, access to patients' medical notes remains limited to most community pharmacists in the USA [100]. In the UK, pharmacists were only given access to patients' summary care records in 2015 [101].

The majority of the interventions in the clinical trials were conducted by pharmacists with a minimum of three years of experience [75–82, 87]. Thus, there are uncertainties as to whether pharmacists with limited experience can produce similar clinical outcomes in practice settings. Another issue to consider is that although medication management services are accepted and being practised in many countries, pharmacists are still burdened with dispensing workloads, inadequate staff, and lack of time in carrying out services [102–104].

### 3.5. Long Term Outcomes

The duration of most diabetes intervention studies ranges between three to 14 months. The improvement in clinical outcomes could therefore only be evaluated over a short-term period. As the main goal in diabetes management is prevention of diabetes related complications such as nephropathy and CVD, there is a need for a long term study incorporating multifactorial interventions. One RCT study of 150 patients followed up for seven years in China [12] found that diabetes nephropathy can be delayed and macrovascular disease can be prevented using tightly controlled BP, cholesterol, and glycaemic targets as defined in USA guidelines. However this study was carried out in a hospital rather than a community setting. As such it remains uncertain if these methods can be translated into primary healthcare settings.

### 3.6. Opportunities for Pharmacists in Preventing Diabetes Complications

In comparison to other healthcare professionals involved in diabetes care, pharmacists are better qualified in pharmacology of medicines and assessment of medication-related problems. In contrast to GPs, community pharmacists are easily accessible to the public with extended opening hours and without the need for prior appointments. They are therefore able to provide medication review services to most people with diabetes. Undoubtedly, pharmacists play an important role and therefore should take on a bigger role in providing diabetes care and further ease the burden off general practitioners.

In order to support pharmacists in delivering consistent quality diabetes care that addresses all the seven evidence-based factors, a structured intervention method may be beneficial. This could take the form of a tool with support materials, checklists, or structured interview questions. Currently there are no published studies on the effectiveness of a standardised and structured method for pharmacists delivering diabetes management.

## 4. Conclusion

This narrative review has highlighted the seven evidence-based factors involved to prevent or delay diabetes related complications and achieve target therapy outcomes. While our findings identified a lack of consistent and systematic multifactorial evidence-based approaches in delivering diabetes care, it did demonstrate pharmacists' contributions towards improving clinical and QOL outcomes. This review has revealed some questions in need of further investigations, in particular, the impact of pharmacists' interventions on all seven evidence-based factors and the effect of long term clinical and health related QOL outcomes.

## Figures and Tables

**Table 1:** Summary of landmark diabetes trials.

Trials	Number of patients	Country (ethnicity)	Measure	Outcome
UKPDS	5102	UK	(1) Intensive blood glucose control using metformin (to achieve HbA1c of 7%) versus conventional treatment. Patient followed up for median of 10.7 years (2) Intensive BP control (less than 150/85 mm Hg) (3) Efficacy of captopril or atenolol as antihypertensive and in controlling microvascular and macrovascular complications	(1) A reduction of 1% in HbA1c^#^ produced significant risk reduction (12%) for any diabetes related end point, 25% risk reduction for microvascular end points, 21% risk reduction for retinopathy and 33% risk reduction for albuminuria at 12 years, and 16% risk reduction for myocardial infarction (2) Significant effect on microvascular and macrovascular complications (3) Captopril and atenolol were equally effective antihypertensives in preventing microvascular and macrovascular complications

ADVANCE	10000	20 countries from Asia, Europe and North America, and Australia	Intensive lowering of blood glucose to HbA1c of 6.5% (gliclazide modified release) in addition to other therapies and BP (perindopril/indapamide combination) compared to UKPDS trial Median follow-up of 5 years	(1) Significant reduction in microvascular events (2) Severe and minor hypoglycemia more frequent in intensive arm (3) Hospitalisation more frequent in intensive arm

DCCT	1441	USA and Canada	Intensive therapy using three or more daily injections compared to conventional treatment (one or two insulin injections daily) among type 1 diabetes patients Mean follow-up of 6.5 years	(1) Intensive therapy reduced microalbuminuria: 39%, albuminuria: 54%, neuropathy: 60%, progression of retinopathy: 54%, and risk of retinopathy: 76% (2) Significant weight gain and diabetic ketoacidosis were reported more on intensive arm

ACCORD^*∗*^	(1) 10251 (2) 4733 (3) 5518	USA and Canada	(1) Intensive intervention to control hyperglycemia to less than HbA1c of 6.0% (2) Two targets for systolic levels in BP control (<120 versus <140) (3) Two regimens for plasma lipid levels. Fenofibrate and simvastatin versus simvastatin alone Mean follow-up of 3.4 years	(1) All cause mortality was significantly greater in the intensive arm (2) No reduction in macrovascular, mortality, or myocardial infarctions (3) No significant difference between the two arms

VADT	1791	USA	Comparison between intensive and standard glucose control Mean follow-up of 5.6 years	(1) No significant difference in the rates of CVD events, death, or microvascular complications (2) More hypoglycemia in intensive group

Note: ^#^HbA1c (glycated hemoglobin) reflects average plasma glucose over the previous eight to 12 weeks. It is used as a marker for diabetes control [19].

^*∗*^The ACCORD trial is divided into three different groups of patients, namely, the glycemic, lipid, and blood pressure groups.

**Table 2:** RCT studies led by healthcare professionals other than pharmacists grouped together according to type of interventions.

	Author, year	Study duration (months)	Country	Group size (usual care versus intervention)		Intervention strategy	Results
1	Barrera et al., 2012 [69]	12	USA	138	142	Culturally adapted diabetes intervention	Improvement in sources for dietary practice, problem solving, and physical activity

2	Farmer et al., 2012 [63]	5	UK	81	114	Intervention on adherence, reinforcement of positive belief by nurse	Percentage of adherence days in intervention group was 77.4 and usual care group was 69%

3	Keogh et al., 2011 [65]	6	Ireland	61	60	Motivational interviewing	Significant lower A1C Levels (0.66%), significant improvements in beliefs about diabetes, psychological well-being, diet, exercise, and family support

4	DePue et al., 2013 [59], Sinclair et al., 2013 [62], Spencer et al., 2011 [60]	3–12	American Samoa, Native Hawaiian, and Pacific People	34–134	48–134	Community nurse intervention on self-management among diabetes patients	Significant reduction in HbA1c (0.5%–1.1%), understanding of diabetes self-management, and performing diabetes self-management

5	Fischer et al., 2012 [67]	20	USA	381	381	Nurses independently initiated and titrated lipid therapy and promoted behavioural change through motivational interviewing and self-management techniques	Percentage of patients achieving target LDL increased in intervention group

6	Williams et al. 2012 [71], Quinn et al., 2011 [70]	6–12	Australia and USA	60–82	60–81	Nutrition, blood glucose monitoring, medication taking, and lifestyle through telephone	Significant improvement in HbA1c (0.8%–1.9%) and health related quality of life

7	Kang et al., 2010 [66]	6	USA and Taiwan	28	28	Psychological family intervention by healthcare professionals (nurse, pharmacist, physician, physiotherapist, dietitians, foot therapist, and social workers)	Statistically significant improvements in HbA1c (1.35%), beliefs about diabetes, psychological well-being, diet, exercise, and family support

8	Chen et al., 2012 [64]	3	Taiwan	111	104	Motivational interview using Miller and Rollnick's (2002) approach. Intervention based on readiness to change	Improvement in self-management, self-efficacy, quality of life, and HbA1c (0.8%)

9	Wu et al., 2011 [61]	6	Taiwan	73	72	Self-management programmes by nurses	The scores for efficacy expectations, outcome expectations, and self-care activities had significantly increased in the intervention group at the 3- and 6-month follow-ups

10	Adachi et al., 2013 [41]	6	Japan	93	100	Dietician in primary care	Increased intake of vegetable and reduced intake of mean energy intake and HbA1c reduction of 0.7%

11	Weinger et al., 2011 [68]	12	USA	96 & 92	94	Nurse and dietician trained to use brief behavioural cognitive strategies	Improvements in reduction of HbA1c to 0.8%

12	Yang et al., 2013 [12]	84 (7 years)	China	68	70	Diet, exercise, BP, cholesterol, and glycaemic by endocrinologist in hospital	Reduction in macrovascular outcomes

**Table 3:** Pharmacist led diabetes intervention RCT studies grouped together according to types of intervention.

	Country	Duration (month)	Group size control versus intervention		Types of intervention	Pharmacist participants	Results
1	Pakistan [75]	5	170	178	Education and glycaemic control	Clinical pharmacists with minimum experience of 3 years in hospital setting	Reduced body mass index and waist circumference, fasting blood glucose, and HbA1c (−1.01%). Increase in compliance, foot care, and SMBG

2	Nigeria and Hong Kong [76, 77]	9–12	54–110	51–110	Education, lifestyle, and medication	Experienced hospital pharmacists	Improved quality of life significant reduction in CVD risk, HbA1c levels (1.57%), LDL, and increased level of medication understanding

3	Brazil, Jordan, Belgium, and USA [78, 80, 82, 85–88]	6–14	23–2303	23–1797	Education and medication	Community pharmacists with minimum of 4 years of experience in diabetes management, board-certified pharmacotherapy specialists trained in diabetes	Significant reduction of HbA1c (0.5%–1.6%), FBG, total cholesterol, LDL cholesterol, TGL, BP and increase in HDL, improvement in self-management, and medication adherence

4	Iran [47]	3	87	87	Education	Clinical pharmacists	Improvements in FBG and HbA1c (1.7%)

5	USA [89]	1	39	33	Medication	Clinical pharmacists with 2 years' experience	No significant difference in HbA1c, LDL, and BP

6	USA [90]	4	28	28	Medication	Clinical pharmacists	Significant improvement in HbA1c (0.9%)

7	Malaysia and USA [79, 91]	9–12	42–201	43–195	Glycaemic control, BP, cholesterol, CVS risk, education, lifestyle, and medication	Experienced clinical pharmacists trained as diabetes pharmacists	Significant improvement in HbA1c (1.7–1.8% reduction) and medication adherence levels

8	Canada [81]	12	93	102	Medication, BP, cholesterol, and glycaemic	Community pharmacists certified as diabetes educators with >5 years of practice experience	Reduction in Framingham risk score, 1.2%

9	USA [84]	3	24	19	Education, lifestyle, and medication	Community pharmacists trained as diabetes pharmacists	Reduction in HbA1c of 0.93% and mean body mass index

10	USA [83]	6	49	50	Medication and behavioural interventions	Community pharmacist certified as diabetes educators	Significant improvements in exercise, foot care, HbA1c (0.41%), LDL, and BP

11	Hawaii [92]	7	62	128	Medication and life coach counselling	Community pharmacists trained as diabetes pharmacists	Significant effect on QOL and body mass index

12	USA [93]	6	24124	5123	Statin, ACE/ARB initiation, and total days of medication supply per month (adherence)	Community pharmacists trained to deliver intervention	Increased adherence and GP initiation of ACE/ARB and statin
